# Bayesian techniques for analyzing group differences in the Iowa Gambling Task: A case study of intuitive and deliberate decision-makers

**DOI:** 10.3758/s13423-017-1331-7

**Published:** 2017-07-06

**Authors:** Helen Steingroever, Thorsten Pachur, Martin Šmíra, Michael D. Lee

**Affiliations:** 1 Department of Psychology, University of Amsterdam, PO Box 15906, 1001 NK, Amsterdam, The Netherlands; 2 Center for Adaptive Rationality, Max Planck Institute for Human Development, Berlin, Germany; 3 Masaryk University, Brno, Czech Republic; 4 University of California, Irvine, CA USA

**Keywords:** Cognitive modeling, Reinforcement learning models, Bayes factors, Product space method, Latent-mixture modeling

## Abstract

The Iowa Gambling Task (IGT) is one of the most popular experimental paradigms for comparing complex decision-making across groups. Most commonly, IGT behavior is analyzed using frequentist tests to compare performance across groups, and to compare inferred parameters of cognitive models developed for the IGT. Here, we present a Bayesian alternative based on Bayesian repeated-measures ANOVA for comparing performance, and a suite of three complementary model-based methods for assessing the cognitive processes underlying IGT performance. The three model-based methods involve Bayesian hierarchical parameter estimation, Bayes factor model comparison, and Bayesian latent-mixture modeling. We illustrate these Bayesian methods by applying them to test the extent to which differences in intuitive versus deliberate decision style are associated with differences in IGT performance. The results show that intuitive and deliberate decision-makers behave similarly on the IGT, and the modeling analyses consistently suggest that both groups of decision-makers rely on similar cognitive processes. Our results challenge the notion that individual differences in intuitive and deliberate decision styles have a broad impact on decision-making. They also highlight the advantages of Bayesian methods, especially their ability to quantify evidence in favor of the null hypothesis, and that they allow model-based analyses to incorporate hierarchical and latent-mixture structures.

The Iowa Gambling Task (IGT; Bechara et al., 1994) is arguably the most popular neuropsychological paradigm for assessing complex, experience-based decision-making (Toplak et al., 2010). In the IGT, participants are asked to choose successively from four decks. Two of the decks are bad decks, because they result in negative long-term outcomes, while the remaining two decks are good decks, because of their positive long-term outcomes. Successful performance hinges on initially exploring all of the decks and then moving to the two good decks. There is considerable evidence that the IGT performance of healthy decision-makers (i.e., participants who do not have any neurological impairments) differs from that of clinical populations, such as patients with lesions to the ventromedial prefrontal cortex (Bechara et al., 1998; Bechara et al., 1999; Bechara et al., 2000), pathological gambling (Cavedini et al., 2002), obsessive–compulsive disorder (Cavedini et al., 2002), psychopathic tendencies (Blair et al., 2001), or schizophrenia (Bark et al., 2005; Martino et al., 2007).

To compare groups in IGT performance, these studies have mainly relied on an analysis of the proportion of choices from the good decks as compared to the bad decks, with conclusions based on frequentist techniques, such as *t* tests and analyses of variance (ANOVAs). In addition, to investigate the psychological processes that underlie people’s performance, several reinforcement-learning (RL) models have been proposed. These models assume that card selection on the IGT results from an interaction between distinct psychological processes including motivation, memory, and response consistency (Busemeyer et al., 2003). Using these models, it has been possible to reveal group differences in cognitive processes despite an absence of group differences in IGT choices (e.g., Yechiam et al., 2008). Popular RL models for IGT data are the Expectancy Valence model (EV; Busemeyer & Stout, 2002; Yechiam et al., 2008) and the Prospect Valence Learning model (PVL; Ahn et al., 2008; Ahn et al., 2011; see Steingroever et al., 2013a, for additional references and a detailed description of the EV and PVL models). More recently, it has been shown that a hybrid version of the EV and PVL models—the PVL-Delta model—outperforms the EV and PVL model in many model comparison analyses (Ahn et al., 2008; Fridberg et al., 2010; Steingroever et al., 2013b; Steingroever et al., 2014, but see also Worthy et al., 2013, for the Value-Plus-Perseveration model, and Dai et al., 2015, for the PVL2 model).

The current standard approach for comparing model parameters between groups is to (1) estimate the parameters for each participant separately using maximum likelihood, (2) average the individual point estimates to obtain a group estimate, and (3) use frequentist statistical tests, such as independent-samples *t*-tests, Jonckheere–Terpstra tests, or Mann–Whitney *U* tests, to compare the estimates across groups (e.g., Cella et al., 2012; Escartin et al., 2012; Yechiam et al., 2008). This approach, however, has several limitations. First, individual-level maximum likelihood results in less precise and less stable parameter inferences compared to Bayesian hierarchical parameter estimation (Ahn et al., 2011; Scheibehenne & Pachur, 2015; Shiffrin et al., 2008; Wetzels et al., 2010). Second, the group averaging procedure risks underestimating the variability of the group estimate because individual parameter estimates, which often have high variance, are integrated into a group average that has a much lower variance than the individual point estimates (Wetzels et al., 2010). Third, the group averaging procedure ignores commonalities across participants of the same group (Wetzels et al., 2010). Fourth, and more generally, there are several well-known problems inherent with frequentist tests, such as *p* values overstating the evidence against the null hypothesis (Berger & Delampady, 1987; Edwards et al., 1963; Johnson, 2013; Sellke et al., 2001), classical hypothesis testing not being able to quantify evidence in favor of the null hypothesis, and frequentist sequential testing, compared to Bayesian sequential testing (Rouder 2014), being much less flexible, since it requires researchers to specify in advance the total duration of the data collection period (e.g., Reboussin et al., 2000) and the number of interim analyses (e.g., Pocock, 1977).^1^

Here, we present a Bayesian approach to examine whether two groups differ in their IGT performance, encompassing both behavioral and model-based analyses. We illustrate our Bayesian approach by comparing IGT performance of decision-makers who report preferring an intuitive (affective) decision style and those preferring a deliberate (planned) decision style. Based on existing self-report instruments, a relationship between decision style and decision performance has been demonstrated (Phillips et al., 2016). It is currently unclear, however, to what extent this also holds for the IGT. A comparison of IGT performance of people with intuitive versus deliberate decision styles seems particularly interesting because the prominent somatic marker hypothesis (Bechara et al., 1997; Damasio et al., 1991; Damasio, 1994) suggests that intuitive, affective processes may be of particular importance for successful performance on the IGT. To conduct such a group comparison, we apply a Bayesian repeated-measurement ANOVA and illustrate three complementary cognitive analyses for comparing the groups on parameters estimated with the PVL-Delta model: (1) Bayesian hierarchical parameter estimation, (2) Bayes factor model comparison, and (3) Bayesian latent-mixture modeling (see also Lee et al., 2015). All our analyses were conducted using JASP (JASP Team 2015), R (R Core Team, 2015), and the Stan software (Hoffman & Gelman, 2014; Stan Development Team, 2014a, b, c), all of which are freely available. We make the relevant R and Stan code available online, and it can be adapted for similar IGT models and similar decision-making tasks.

The outline of this article is as follows. The next section describes the IGT, the PVL-Delta model, and its Bayesian hierarchical implementation, together with a brief review of Bayesian statistics. The following sections then present the proposed methodology and its application to IGT data of intuitive and deliberate decision-makers. In the final section, we summarize our findings and discuss the methodological contribution of our proposed analysis approach and implications for the notion of intuitive and deliberate decision styles.

## The IGT and PVL-Delta model

### The IGT

In the standard version of the IGT, participants are initially given $2000 (hypothetically) and are presented with four decks of cards with different payoffs (see also Steingroever et al., 2013; Steingroever et al., 2013a; Steingroever et al., 2013b; Steingroever et al., 2014; Steingroever et al., 2016). Participants are instructed to choose, over several rounds, cards in order to maximize their long-term net outcome (Bechara et al., 1994; Bechara et al., 1997). Unbeknownst to the participants, the task has a fixed number of (typically) 100 trials. After each card selection, participants receive feedback on the rewards and losses (if any) associated with that card, as well as their running tally of rewards and losses over all trials so far.

A crucial aspect of the IGT is to what extent participants eventually learn to prefer the good decks over the bad decks, because only choosing from the good decks maximizes their long-term net outcome. The good decks are typically labeled as decks C and D, whereas the bad decks are labeled as decks A and B. Table 1 presents a summary of the common payoff scheme as developed by Bechara et al. (1994). This table illustrates that decks A and B yield high constant rewards, but even higher unpredictable losses: hence, the long-term net outcome is negative. Decks C and D, on the other hand, yield low constant rewards, but even lower unpredictable losses: hence, the long-term net outcome is positive. In addition to having different payoff magnitudes, the decks also differ in the frequency of losses: decks A and C yield frequent losses, while decks B and D yield infrequent losses.

**Table 1:** Summary of the payoff scheme of the traditional IGT as developed by Bechara et al. (1994)

	Deck A	Deck B	Deck C	Deck D
	Bad deck with frequent losses	Bad deck with infrequent losses	Good deck with frequent losses	Good deck with infrequent losses
Reward/trial	100	100	50	50
Number of	5	1	5	1
losses/10 cards				
Loss/10 cards	−1250	−1250	−250	−250
Net outcome/10 cards	−250	−250	250	250

### The PVL-Delta model

The PVL-Delta model formalizes people’s performance on the IGT through the interaction of four parameters that have natural psychological interpretations as representing different psychological processes (Ahn et al., 2008; Fridberg et al., 2010; Steingroever et al., 2014; see also Steingroever et al., 2013b; Steingroever et al., 2016). The first assumption of the PVL-Delta model is that, after choosing a card from deck *k* ∈{1,2,3,4} on trial *t*, people evaluate the net outcome associated with the card and this evaluation can be described by the utility function from prospect theory (Tversky & Kahneman, 1992). Formally, the utility is given by

1 $$ \displaystyle u_{k}(t) = \left\{\begin{array}{c l} X(t)^{A} & \text{if}\text{ \(X(t) \geq 0\)} \\ -w \cdot |X(t)|^{A} & \text{if}\text{ \(X(t) < 0\).} \end{array} \right. $$

In this equation, *X*(*t*) represents the net outcome on trial *t*, which is the sum of the experienced reward and loss (i.e., *X*(*t*) = *W*(*t*) −|*L*(*t*)|). The prospect utility function contains the first two model parameters, namely the loss aversion parameter *w* ∈ [0,5], and the outcome sensitivity parameter *A* ∈ [0,1].

The loss aversion parameter *w* quantifies the relative weight of net losses relative to net gains in people’s evaluation of the net outcome of a given card. A value of *w* greater than one indicates a larger impact of negative than of positive net outcomes, whereas a value of *w* approaching one indicates a similar impact of negative and positive outcomes. As *w* approaches zero, negative net outcomes are neglected.

The outcome sensitivity parameter *A* quantifies the extent to which the subjective utility corresponds to the actual net outcome, *X*(*t*). As *A* approaches one, the subjective utility *u*_*k*_(*t*) increases in proportion to the actual net outcome. For values of *A* smaller than one, there is less differentiation between different net outcomes. As *A* approaches zero, the sensitivity to differences in the net outcomes continues to decrease towards the limit in which there is no sensitivity at all.

The PVL-Delta model also assumes that, having formed the utility of the card as described in Eq. 1, people update their expected utility of the just-chosen deck, but keep the expected utilities of the remaining decks unchanged. This updating process is formalized by the delta learning rule:

2 $$ Ev_{k}(t) = Ev_{k}(t - 1) + \delta_{k}(t) \cdot a \cdot (u_{k}(t) - Ev_{k}(t - 1)), $$

where *δ*_*k*_(*t*) is an indicator variable that equals 1 if deck *k* is chosen on trial *t* and otherwise zero. The delta learning rule states that the expected utility of the chosen deck *k* is adjusted upward if the experienced utility *u*_*k*_(*t*) is higher than expected. If the experienced utility *u*_*k*_(*t*) is lower than expected, the expected utility of deck *k* is adjusted downward.^2^This updating process is influenced by an updating parameter *a* ∈ [0,1]. This parameter expresses the memory for past expectancies. A value of *a* close to zero indicates slow forgetting and weak recency effects, whereas a value of *a* close to one indicates rapid forgetting and strong recency effects.

In the next step, the PVL-Delta model assumes that the expected utilities of each deck guide people’s choices on the next trial. This assumption is formalized by the softmax choice rule, also known as the ratio-of-strength choice rule (Luce 1959):

3 $$ P[S_{k}(t + 1)] = \frac{e^{\theta \cdot Ev_{k}(t)}}{{\sum}^{4}_{j=1}e^{\theta \cdot Ev_{j}(t)}}. $$

The PVL-Delta model uses this rule to compute the probability of choosing each deck on each trial. The softmax choice rule includes a sensitivity parameter *𝜃* that controls the extent to which trial-by-trial choices match the expected deck utilities. Values of *𝜃* close to zero indicate random choice behavior (i.e., strong exploration), whereas large values of *𝜃* indicate choice behavior that is strongly determined by the expected utilities (i.e., choices strictly follow the expectancies of the decks).

According to the PVL-Delta model, the sensitivity parameter *𝜃* depends on the final model parameter, the response consistency *c* ∈ [0,5], as follows:

4 $$ \theta = 3^{c}-1. $$

Small values of *c* lead to a small values of sensitivity *𝜃* and thus to more random choices, whereas large values of *c* lead to larger values of *𝜃*, and thus to more deterministic choices.

In summary, the PVL-Delta model has four parameters: (1) an outcome sensitivity parameter *A*, which determines the shape of the utility function, (2) a loss aversion parameter *w*, which quantifies the weight of net losses over net rewards, (3) an updating parameter *a*, which determines the memory for past expectancies, and (4) a response consistency parameter *c*, which determines the balance between exploration and exploitation in the deck choices.

### Bayesian hierarchical implementation of the PVL-Delta model

For our modeling analyses, we used a Bayesian hierarchical implementation of the PVL-Delta model. This implementation assumes that, within each group, probit-transformed model parameters of each participant are drawn from group-level normal distributions characterized by mean and standard deviation parameters: $z_{i}^{\prime } \sim \mathrm {N}\bigl (\mu _{z^{\prime }},\sigma _{z^{\prime }}\bigr )$. Note that we use *z*_*i*_ to refer to a specific PVL-Delta model parameter of participant *i* (i.e., *z*_*i*_ ∈{*A*_*i*_, *w*_*i*_, *a*_*i*_, *c*_*i*_}), and $z_{i}^{\prime }$ to refer to its probit-transformed version (i.e., $z_{i}^{\prime } = {\Phi }^{-1}(z_{i}$) with Φ^−1^ being the inverse of the cumulative standard normal distribution function). In addition, note that parameters with ranges different to the [0,1] interval were transformed to this interval before the analysis, and were only transformed back to their original ranges after the analysis. We assigned a standard normal prior to the group-level means $\mu _{z^{\prime }}$, and a uniform prior ranging from 0 to 1.5 to each group-level standard deviation parameter $\sigma _{z^{\prime }}$ (see Steingroever et al., 2013b, for more details on the implementation, and Wetzels et al., 2010, for the same model specification in the case of the EV model). In this way, the Bayesian hierarchical framework naturally incorporates both differences and commonalities between and within the participants of one group, and produces both inferences about individual-level and group-level parameters (Horn et al., 2015; Lejarraga et al., 2016; Navarro et al., 2006; Rouder & Lu, 2005; Rouder et al., 2005; Rouder et al., 2008). To test our implementation of the PVL-Delta model, we ran several parameter-recovery analyses. The results of two such analyses, indicating good recovery performance, are presented in the appendix of Steingroever et al. (2013b).

Bayesian methods differ from frequentist methods in how they address the two basic goals of statistical inference: parameter estimation and model selection. In Bayesian parameter estimation, inferences about a parameter are based on the posterior distribution of the parameter values given the observed data. A posterior distribution expresses the uncertainty about the value of a parameter based on the modeling assumptions and the observed data. In a Bayesian framework, the so-called Bayes factor is used to quantify the relative probability of the data under two competing models or hypotheses (Berger & Mortera, 1999; Edwards et al., 1963; Jeffreys, 1961; Kass & Raftery, 1995; Rouder et al., 2012; Rouder et al., 2009; Wagenmakers, 2007; Wagenmakers et al., 2010; Wetzels et al., 2009). In particular, BF_01_ quantifies the probability of the data under the null hypothesis (H_0_) relative to the probability of the data under the alternative hypothesis (H_1_). A Bayes factor can, for example, be used to quantify the evidence that the data provide for a model that assumes differences in the loss aversion parameter across two groups of decision-makers ($\mathcal {M}_{1}$), compared to a model that assumes no differences ($\mathcal {M}_{0}$). If, for example, it was found that BF_01_ = 10, this would indicate that the data were ten times more likely under $\mathcal {M}_{0}$ than under $\mathcal {M}_{1}$. To classify the evidential strength of BF_01_ = 10, the Bayes factor categories of Jeffreys (1961) can be used (see also Lee & Wagenmakers, 2013). Accordingly, BF_01_ = 10 is classified as strong evidence for model $\mathcal {M}_{0}$. Alternatively, if it was found that BF_01_ = 1/10, this would indicate that the data were ten times more likely under $\mathcal {M}_{1}$ than under $\mathcal {M}_{0}$. Note that BF_01_ = 1/10 is equivalent to BF_10_ = 10, where the reversed model comparison is expressed by the subscripts of BF. As these possibilities make clear, in contrast to frequentist methods, Bayes factors allow for a quantification of the evidence for the null hypothesis or null model (e.g., Rouder et al., 2009).

## Proposed methodology for comparing groups on the IGT

The IGT has often been used to investigate group differences in decision-making. It is well suited for this goal because it is assumed to tap into a broad spectrum of psychological processes, such as motivation, memory, and response consistency. By comparing group differences in performance—and, in particular, by decomposing the decision behavior using cognitive modeling—there is the potential to identify which processes are different and which are the same across groups of decision-makers. Yechiam et al. (2008), for example, studied the IGT performance of six groups of criminals and a group of healthy participants. They found that even though the six groups of criminals showed similar behavior on the task, the similar (aggregate) choice patterns were produced by different psychological processes. Drug and sex offenders, for instance, over-weighted potential gains as compared to losses, whereas assault criminals tended to make less consistent choices and to focus on immediate outcomes. These findings required the use of a cognitive model because basic behavioral data analyses of the card selection behavior only allow for inferences about the overt choice behavior (see also Wood et al., 2005). These findings thus illustrate that cognitive models help us to gain a deeper understanding of psychological processes relevant to decision-making.

In the remainder of this section, we elaborate on previous efforts to compare IGT performance of two groups by presenting Bayesian state-of-the-art methods for this purpose. We start with a standard method for behavioral data analysis, before proposing a novel set of complementary approaches for cognitive modeling. All approaches will then be applied to data from two groups that are distinguished based on their self-reported decision style.

### Bayesian behavioral data analyses

Basic behavioral data analyses are usually based on general linear models. A standard IGT experiment involves repeated measures for a number of participants in two or more groups over two or more blocks of trials. Accordingly, a Bayesian block-by-block repeated-measures ANOVA on the choices from the good decks (i.e., decks C and D) is appropriate. These sorts of ANOVA analyses can be conveniently performed in JASP (JASP Team, 2015), which is a user-friendly free software with a graphical user interface for conducting Bayesian data analyses. For our analyses, we use the default prior distributions implemented in JASP, that is, Cauchy(0.5) and Cauchy(1) priors for the fixed effects (i.e., block and group) and random effects (i.e., subject), respectively (Rouder et al., 2012).

### Bayesian cognitive modeling analyses

We implemented all of our proposed model-based analyses using Stan (Stan Development Team, 2014a; Stan Development Team, 2014b; Hoffman & Gelman, 2014; see chapter 9 of Stan Development Team, 2014c, for a description on how to implement mixture models in Stan).

#### **Bayesian hierarchical parameter estimation**

The first model-based analysis involves inferring the posterior distributions of the group-level mean parameters for each group independently. These inferences were made using the Bayesian hierarchical implementation of the PVL-Delta model introduced earlier. To assess the account of the PVL-Delta model to the data we used the post hoc fit method. The post hoc fit method compares so-called postdictions to the observed choices. The postdictions are obtained as follows: For a specific participant and a given trial, the parameter estimates of that participant and all information about the choices and associated payoffs on all trials up to the given trial are used to predict the choice on the next trial. This procedure is realized for all trials and for each participant (for more details see Steingroever et al., 2014).

#### **Bayes factor model comparison**

The second model-based analysis involves comparing the group-level mean parameters of the PVL-Delta model across two groups. This is achieved by comparing a model specification that assumes differences in at least one group-level mean parameter across the two groups to a model that assumes no differences in the group-level parameters (i.e., a null model). Since the PVL-Delta model has four parameters of interest, 15 comparisons of this type are required.^3^

When we refer to a model that assumes differences in at least one group-level mean parameter, we index $\mathcal {M}$ by the corresponding group-level mean parameter. $\mathcal {M}_{\mu _{w}\mu _{c}}$, for example, refers to the model that assumes differences in the group-level mean parameter of the loss aversion parameter *w* and of the consistency parameter *c* (i.e., *μ*_*w*,1_≠*μ*_*w*,2_ and *μ*_*c*,1_≠*μ*_*c*,2_, where the second index refers to the group), but no differences in group-level mean parameter of the outcome sensitivity parameter *A* and of the updating parameter *a* (i.e., *μ*_*A*,1_ = *μ*_*A*,2_ and *μ*_*a*,1_ = *μ*_*a*,2_).

For all model comparisons, we assumed that the group-level standard deviation is the same across the two groups (i.e., *σ*_*A*,1_ = *σ*_*A*,2_, *σ*_*w*,1_ = *σ*_*w*,2_, *σ*_*a*,1_ = *σ*_*a*,2_, and *σ*_*c*,1_ = *σ*_*c*,2_). To quantify the relative evidence that the data provide for each of the 16 models, we used Bayes factors assuming equal prior model probabilities for all models. Under this assumption, the Bayes factor BF_01_ simplifies to the posterior model odds $\text {BF}_{01} = p(\mathcal {M}_{0} | D) / p(\mathcal {M}_{1} | D)$, that is, the ratio of the posterior probability of model $\mathcal {M}_{0}$ relative to the posterior probability of model $\mathcal {M}_{1}$. The posterior probability of a specific model $\mathcal {M}$ was estimated by means of the product space method (Carlin & Chib, 1995; Lodewyckx et al., 2011. This method is based on the construction of a “supermodel” that implements a hierarchical combination of the models to be compared. The hierarchical combination is achieved by a model index vector that, on a given sample, takes on a value indexing the model that is visited on that sample to account for the observed data. The posterior probability of a model under consideration is then given as the proportion of times that that model is visited to account for the observed data (see Appendix B for more details on the product space method).

We conducted several tests to establish the stability of the Bayes factor estimates. First, we confirmed good sampling behavior of the model indicator variable *z* (i.e., good mixing and low autocorrelations, that is, frequent model switches; Lodewyckx et al., 2011). Secondly, we repeated the product space method with fewer iterations (i.e., 5000 samples instead of 7000 of each chain after having discarded the first 1000 samples of each chain as burn-in). The stability of the Bayes factor estimates was confirmed because the difference in corresponding estimated posterior model probabilities was smaller than 0.01 and the Bayes factors of both analyses resulted in the same qualitative conclusions (i.e., using the classification scheme of Jeffreys, 1961, corresponding Bayes factors of both runs were classified into identical evidence categories). Third, the our Stan model file was discussed on the Stan users mailing list.^4^

#### **Bayesian latent-mixture modeling**

The first two model-based analyses focus on parameter estimation and model selection, respectively. Though relatively standard approaches in the general Bayesian statistics literature, they are not routinely applied in the context of the IGT and associated cognitive modeling. The third model-based analysis, which combines elements of parameter estimation and model selection in a complementary way, is novel both in the context of the IGT and in Bayesian applications more generally. This analysis involves a two-group latent hierarchical mixture model (Lee et al., 2015; chapter 6 in Lee and Wagenmakers, 2013).^5^

For the first two model-based analyses, we considered two separate data sets (in our example below, the first data set consists of deliberate decision-makers, whereas the second data set consists of intuitive decision-makers). For the latent-mixture analysis, in contrast, we consider all of the participants in a single data set and ignore the knowledge about each participant’s true group membership. However, we continue to assume that each participant comes from one of two groups, but it is thus unknown which group each participant comes from. The goal of the latent-mixture modeling is then to examine whether the correct group membership for each participant can be inferred from their behavior on the IGT.

Formally, in the two-group case, group membership is indexed by a binary indicator variable *z*_*i*_, so that *z*_*i*_ = 0 and *z*_*i*_ = 1 indicate that the *i*-th participant belongs to the first and second group, respectively. The prior for these indicator parameters is *z*_*i*_ ∼ Bernoulli(*ψ*) with *ψ* ∼ Uniform(0, 1). Consequently, *ψ* corresponds to the base rate of membership to the second group. This choice of priors means that each participant is *a priori* equally likely to be assigned to either group. The latent-mixture model analysis yields the probability for each individual participant to belong to each of the groups, as well as a posterior distribution for the base rate.

One way to apply this latent-mixture analysis is to use the same priors for model parameters as used in the first cognitive-modeling analysis (i.e., the Bayesian hierarchical parameter estimation). In this case, the inferences made by the latent-mixture analysis about the group membership of each participant reflect how people would be classified without any prior knowledge of the true memberships. If these inferred group memberships agree with the actual ones, then the analysis provides strong evidence that the behavioral data and model separate people into the proposed groups.

In this article, we pursue a second, more novel, way to apply the latent-mixture model. Our approach uses highly informative priors, so that each group is defined in terms of group-level parameter inferences based on the true group memberships. These priors approximate the posteriors from the first cognitive-modeling analysis. Formally, within each of the two groups, we assume that the probit-transformed individual-level parameters are drawn from a group-level normal distribution: $z_{i}^{\prime } \sim \mathrm {N}\bigl (\mu _{z^{\prime }},\sigma _{z^{\prime }}\bigr )$. We assigned a normal prior to the group-level means $\mu _{z^{\prime }}$, and a truncated normal prior (allowing for only positive values) to the group-level standard deviations, $\sigma _{z^{\prime }}$. These (truncated) normal prior distributions are characterized by means and standard deviations obtained from the first cognitive-modeling analysis. That is, we use the mean $\bar {x}_{\mu _{z^{\prime }}}$ and the standard deviation $s_{\mu _{z^{\prime }}}$ of the posterior distribution of $\mu _{z^{\prime }}$ obtained from the first cognitive-modeling analysis to specify the prior distribution on $\mu _{z^{\prime }}$ (i.e., $\mu _{z^{\prime }} \sim \mathrm {N}(\bar {x}_{\mu _{z^{\prime }}}, s_{\mu _{z^{\prime }}})$) in this informed latent-mixture model approach, and analogous for the prior distribution on $\sigma _{z^{\prime }}$. This way of constructing the priors produces highly informative priors that approximate the posterior distributions from the first cognitive-modeling analysis. This analysis obviously uses the behavioral data twice—once to construct the prior distributions, and once to fit the latent-mixture model—and so cannot be used to make inferences about model parameters. It does, however, potentially provide a strong test of patterns of group membership. In particular, if the true group memberships of participants cannot be inferred under these ideal conditions, there is strong evidence that the model and data do not distinguish the participants into the proposed groups.

## Case study: Intuitive versus deliberate decision-makers

Whereas many early applications of the IGT focused on comparing clinical to control groups, the task has increasingly also been used to study how individual differences in cognitive abilities (e.g., executive functions, intelligence), mood, age, education, and personality among healthy participants can explain differences in decision-making (Beitz et al., 2014; Buelow & Suhr, 2009; Davis et al., 2008; Suhr & Tsanadis, 2007; Toplak et al., 2010; Wood et al., 2005). One interesting individual difference variable that has recently received much attention is *decision style* (e.g., Phillips et al., 2016). One prominent distinction here is between persons who prefer making decisions using an intuitive decision mode and those who prefer a deliberate decision mode. These two types of decision-makers can be reliably distinguished using scales measuring a person’s self-reported tendency to rely on an intuitive and a deliberate approach when making decisions (Burns & D’Zurilla, 1999; Pacini & Epstein, 1999; Scott & Bruce, 1995). For instance, Betsch (2004) used a self-report inventory to assess people’s tendencies to generally rely on an intuitive, affect-based decision mode (with items such as “I tend to use my heart as a guide for my actions”) and a deliberate, cognition-based decision mode (e.g., “I want to have a full understanding of all problems”). The author found reliable individual differences indicated by high internal validities of the scales (see also Betsch & Iannello, 2010; Pacini & Epstein, 1999).

Differences in decision style might underlie the considerable behavioral heterogeneity often observed in decision-making (e.g., Pachur & Olsson, 2012; Steingroever et al., 2013a). Indeed, there is evidence that self-reported decision style is related to decision behavior. For instance, Schunk and Betsch, (2006) found that when choosing between monetary lotteries, decision-makers with higher scores on the intuition scale showed faster decision times than deliberate decision-makers. In the same task, deliberate decision-makers showed stronger sensitivity to outcome information (indicated by a more linear utility function) than intuitive decision-makers. Finally, when participants were asked to price goods (e.g., coffee mugs), Betsch and Kunz (2008) found that participants who were instructed to operate in either a spontaneous or reflective fashion decided differently depending on whether the instructed decision mode matched their personal decision style. Specifically, under “decisional fit” people priced the objects more positively than under decisional misfit.

A recent meta-analysis by Phillips et al. (2016) found that individual differences in decision styles have a reliable relation to differences in decision performance. The size of the effect and whether an intuitive or a deliberate decision style leads to better performance, however, varies substantially across tasks. Because Phillips et al.’s (2016) meta-analysis mainly encompassed reasoning and judgment tasks, it is currently unclear whether the impact of decision style on decision performance also holds for the IGT. To our knowledge, there is only a single study that has studied the impact of decision style on IGT performance, but this study focused on a deliberate decision style only and found inconsistent results (Harman 2011). It is therefore interesting to investigate the link between decision style and behavior on the IGT more rigorously, including measures of preference of both intuitive and deliberate decision modes and including a decomposition of the behavior with computational modeling (thus disentangling, for instance, motivation and memory processes). After all, as Wood et al. (2005) and Damasio et al. (2008) have shown, the processes underlying behavior on the IGT can differ between groups, as revealed with computational modeling, even if IGT performance itself does not differ across groups.

Moreover, the IGT seems a promising context for studying the impact of decision style because—as has also been noted elsewhere (Dunn et al., 2006; Turnbull et al., 2005)—there is a strong conceptual similarity between the notion of an intuitive decision style and the intuitive, affective processes that are, according to Bechara et al. (1997) and the so-called somatic marker hypothesis (e.g., Damasio et al., 1991; Damasio, 1994), crucial for good IGT performance. The somatic marker hypothesis assumes that people develop from feedback “feelings generated from secondary emotions ... to predict future outcomes of certain scenarios” (Damasio, 1994, p. 174). Patients with lesions to the ventromedial prefrontal cortex, a region in the brain where these somatic markers are assumed to be represented, showed poorer IGT performance than healthy controls (i.e., they made fewer choices from the decks that are profitable on the long run), despite having unimpaired cognitive functioning (Bechara et al., 1997). The patients also showed lower affective responses, indicated by skin conductance responses, before selecting a card from the bad decks. It was argued that healthy participants, but not the patients, had developed affective signals in response to net losses at previous trials, and since these net losses are more frequent and pronounced in the bad decks, this helped the participants to learn to avoid them. These results suggest that the operation of affective, intuitive processes may be an important contributor to successful performance on the IGT (for critical discussions, see Maia & McClelland, 2004; Newell & Shanks, 2014). If so, decision-makers who report to prefer an intuitive decision style, thus paying considerable attention to affective signals when making decisions, might perform better than those who report to prefer a deliberate decision style. This research question is the focus of the following case study—a case study that serves to illustrate our proposed Bayesian methodology.

### Data

**Fig. 1:**
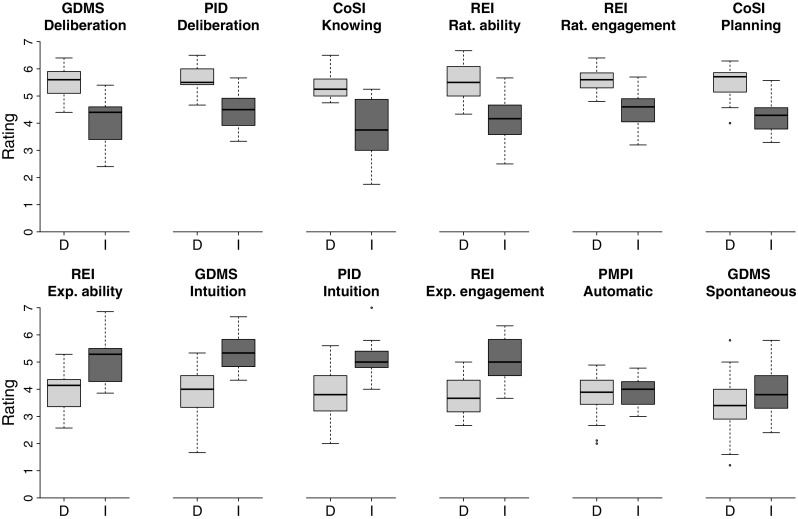
Distribution of scores on the 12 subscales of the questionnaire compiled by Betsch and Iannello (in preparation), separately for the deliberate group (i.e., D) and the intuitive group (i.e., I)

Seventy students from the University of Basel (49 female; average age 24.9 years, *SD* = 5.8, range = 19 − 51 years) participated in the study. Following the administration of a computerized version of the IGT, participants completed a self-report inventory complied by Betsch and Iannello (in preparation) to measure individual participants’ decision style. This inventory consists of 70 items covering a total of 12 subscales (e.g., deliberation, knowing, rational engagement, experiential engagement, spontaneous), taken from various other established instruments measuring intuitive and deliberate decision styles (Betsch, 2004; Burns & D’Zurilla, 1999; Epstein et al., 1996; Scott & Bruce, 1995, e.g., ). For instance, participants indicated their agreement on a seven-point scale to statements such as “When I make a decision, I trust my inner feeling and reactions.” and “The right way to decide usually comes to mind almost immediately.” (intuitive style), and “I like to analyze problems.” and “I usually have clear, explainable reasons for my decisions.” (deliberate style; see Table 6 in Appendix A for a full list of the items used). An overview and a discussion of the internal and construct validity of the subscales is provided by Betsch and Iannello (2010). Cronbach’s Alpha for the subscales based on the current data—showing, overall, rather high internal reliability—are provided in Table 6 of Appendix A. Based on the mean score for each participant on each subscale, we conducted a principal component analysis with a rotation based on the varimax method. The Kaiser criterion suggested a three-factor solution (i.e., a deliberation factor, an intuition factor, and a spontaneity factor). Following previous research (Betsch & Kunz 2008), we classified participants as intuitive if they had both a factor score above the median of the intuition factor and a factor score below the median of the deliberation factor. Participants with the opposite pattern were classified as deliberate. This classification scheme yielded 19 participants in the intuitive group and 19 participants in the deliberate group. Thirty two participants thus remained unclassified and were excluded from the analyses presented in this article (more details can be found in the Appendix A).^6^ Figure 1 uses boxplots to summarize the distribution of scores on the 12 subscales, separately for the intuitive group and the deliberate group. As can be seen, the groups have strongly different profiles on the scales and cover different value ranges.

### Behavioral data analyses

**Fig. 2:**
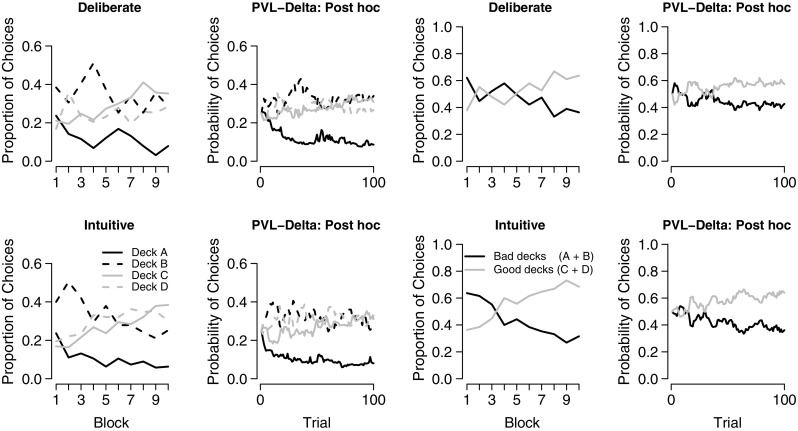
Mean proportion of choices from each deck within ten blocks of both groups of decision-makers (*first column*). Each block contains ten trials. The *second column* shows the predictions of the PVL-Delta model for both groups of decision-makers. The predictions were obtained by computing the mean probabilities of choosing each deck on each trial according the post hoc absolute fit method (see Steingroever et al., 2014). The *third* and *fourth* columns show the same information as the first two columns, respectively, but aggregated across both good and both bad decks

In order to obtain a visual impression of the group-level deck preferences across trials, the first and third columns of Fig. 2 show, separately for intuitive and deliberate decision-makers, the proportion of choices from each deck as a function of ten blocks (see Steingroever et al., 2013, for a discussion of the importance of considering each deck separately and not aggregated across all trials), and the proportion of choices from the good and bad decks, respectively. The figure suggests similar deck preferences for the intuitive and deliberate decision-makers. Specifically, although both groups failed to develop a clear avoidance of bad deck B, overall they learned to make more choices from the good decks than from the bad decks. There appears to be a slight trend for stronger learning in the group of intuitive decision-makers.

We applied our proposed Bayesian data analysis in the form of a 10 (block) x 2 (decision style) repeated measures ANOVA. The results of this analysis are presented in Table 2 and showed that the data are 370506.491/101921.230 = 3.64 times more likely under the “Block model” that assumes an effect of block, but no effect of group than under the “Block + Group model” that assumes both group and block differences (i.e., the Bayes factor BF_01_ is 3.64 in favor of the model that includes no main effect of group). According to the classification scheme of Jeffreys (1961), this can be considered as moderate evidence for the “Block model” as compared to the “Block + Group model”. In addition, the data are about five times more likely under the model that assumes that there is no interaction between block and decision style (but a group and block effect) than under the model that assumes that there is such an interaction effect and group and block effects (i.e., the Bayes factor is 101921.230/18945.710 = 5.38 in favor of the model that includes no interaction effect between block and decision style).^7^ This can also be classified as moderate evidence for the null model (Jeffreys, 1961). These results suggest that deliberate and intuitive decision-makers show similar learning curves on the IGT.

**Table 2:** Output of the Bayesian repeated measures ANOVA conducted in JASP

Model	BF_10_
Null model	1.000
Block	370506.491
Group	0.256
Block + Group	101921.230
Block + Group + Block * Group	18945.710

### Cognitive modeling analyses

Even though the behavioral data analysis suggests that intuitive and deliberate decision-makers show similar deck preferences on the IGT, there might still be group differences in the cognitive processes underlying the decisions (see also Wood et al., 2005; Yechiam et al., 2008). To investigate this possibility, we next decompose the IGT performance of the two groups using three different cognitive modeling analyses.

In each of the three cognitive modeling analyses, we used random starting values for the parameter estimation. For the first two analyses, we ran three Hamiltonian Monte Carlo (HMC) chains, and for the third cognitive modeling analysis we ran five HMC chains. We collected 4000, 7000, and 9000 samples of each chain after having discarded the first 2000, 1000, and 1000 samples of each chain as burn-in in the case of first, second, and third analysis, respectively. Visual inspection of the chains suggested that the samples provided a valid approximation to the joint posterior parameter distribution. This was confirmed by the $\hat {R}$ statistic—a formal diagnostic measure of convergence that compares the between-chain variability to the within-chain variability (Gelman & Rubin, 1992)—because all parameters had $\hat {R}$ values below 1.05. As a rule of thumb, values of $\hat {R}$ close to 1.0 indicate adequate convergence to the stationary distribution, whereas values greater than 1.1 indicate inadequate convergence.

#### **Bayesian hierarchical parameter estimation**

Before interpreting the estimated model parameters, we assessed whether the PVL-Delta model sufficiently accounts for the data of both groups using the post hoc absolute fit method (see Steingroever et al., 2014). The post hoc fit performance of the PVL-Delta model is presented in the second and fourth column of Fig. 2 for each deck separately and aggregated across both good and bad decks, respectively. Comparing the post hoc performance of the model to the data, it is apparent that the PVL-Delta model captures the qualitative choice pattern in both groups. In particular, as the task proceeds, the model predicts that both groups learn to make more choices from the good decks, and that intuitive decision-makers make slightly more choices from the good decks. The PVL-Delta model thus captures key trends in the data for both groups, allowing for meaningful conclusions from the model parameters.

**Fig. 3:**
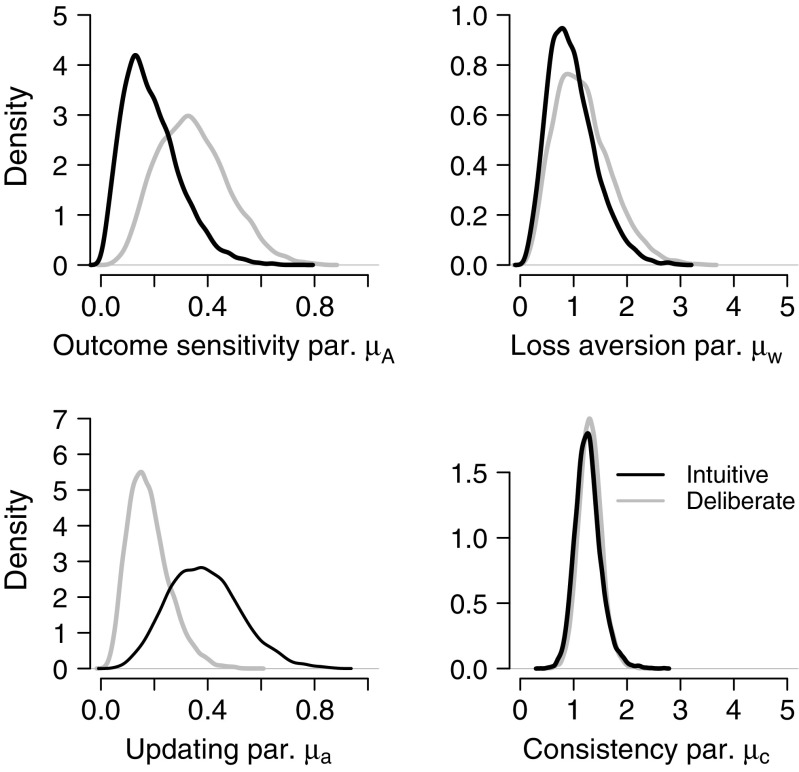
Posterior distributions of the group-level parameters of both groups obtained from fitting the PVL-Delta model to the data of each group separately

Figure 3 shows the posterior distributions of the group-level mean parameters of the PVL-Delta model, separately for the intuitive and the deliberate decisions-makers. The posterior distributions show that deliberate decision-makers tend to have a higher outcome sensitivity parameter *μ*_*A*_ (i.e., a better correspondence between the objective and the subjective utilities of the decks), but a lower updating parameter *μ*_*a*_ (i.e., less forgetting and weaker recency effects) than intuitive decision-makers. In addition, the posterior distributions suggest that the groups differ neither on the loss aversion parameter *μ*_*w*_ nor on the choice consistency parameter *μ*_*c*_. Note that these conclusions are based only on a visual comparison of the posterior distributions.

#### **Bayes factor model comparison**

We next report the results of the Bayes factor model comparison, first discussing the posterior model probabilities, and then deriving Bayes factors according to the formula: $\text {BF}_{\Omega , abcd} = \hat {p}(\mathcal {M}_{\Omega } \mid D) / \hat {p}(\mathcal {M}_{abcd} \mid D)$, that is, the ratio of the estimated posterior model probability of model $\mathcal {M}_{\Omega }$ and $\mathcal {M}_{abcd}$.^8^

**Table 3:** Posterior model probabilities of the null model and models that assume differences in only one group-level mean parameter under the assumption of equal prior model probabilities

$\hat {p}(\mathcal {M}_{\Omega } | D)$	$\hat {p}(\mathcal {M}_{\mu _{A}} | D) $	$\hat {p}(\mathcal {M}_{\mu _{w}} | D)$	$\hat {p}(\mathcal {M}_{\mu _{a}} | D)$	$\hat {p}(\mathcal {M}_{\mu _{c}} | D)$
0.20	0.15	0.07	0.16	0.03

The posterior model probabilities of models that are neither shown in this table nor in Table 4 are less than .05

Tables 3 and 4 show the posterior model probabilities for eight of the models under the assumption of equal prior model probabilities of all models. The posterior model probabilities of the remaining models are below 0.05 and are not shown. The posterior probability of a specific model quantifies the relative plausibility for that model given the prior model probability and the evidence from the data (Berger & Molina, 2005). From the tables it is evident that the null model $\mathcal {M}_{\Omega }$, which assumes no differences between intuitive and deliberate decision-makers in the group-level mean parameters, has the highest posterior model probability. The evidence for the null model is weakest when it is compared to the model that assumes differences between intuitive and deliberate participants in the outcome sensitivity parameter (i.e., model $\mathcal {M}_{\mu _{A}}$; $\text {BF}_{\Omega , \mu _{A}} = 1.36$) and the model assuming differences in the updating parameter (i.e., model $\mathcal {M}_{\mu _{A}}$; $\text {BF}_{\Omega , \mu _{a}} = 1.23$). According to Jeffreys (1961), the evidence for the null model compared to these two models can be characterized as anecdotal. When compared to model $\mathcal {M}_{\mu _{w}}$ (i.e., the model that assumes differences in the loss aversion parameter), the Bayes factor analysis indicates that the data are about three times more likely under the null model ($\text {BF}_{\Omega , \mu _{w}} = 2.84 $); according to Jeffreys (1961), this level of evidence is also anecdotal. In addition, the data provide moderate evidence for the null model compared to model $\mathcal {M}_{\mu _{c}}$ (i.e., the model that assumes differences in the consistency parameter; $\text {BF}_{\Omega , \mu _{c}} = 6.31$). These findings are consistent with Fig. 3, where the largest differences in the posterior distributions were on the group-level mean of the outcome sensitivity parameter and the updating parameter; the group-level means for the loss aversion parameter and the consistency parameter had posterior distributions that are highly overlapping between the intuitive and deliberate decision makers.

**Table 4:** Posterior model probabilities of models that assume differences in two group-level mean parameters under the assumption of equal prior model probabilities

$\hat {p}(\mathcal {M}_{\mu _{A}\mu _{w}} | D)$	$\hat {p}(\mathcal {M}_{\mu _{A}\mu _{a}} | D)$	$\hat {p}(\mathcal {M}_{\mu _{w}\mu _{a}} | D)$
0.05	0.12	0.06

The posterior model probabilities of models that are neither shown in this table nor in Table 3 are less than .05

When comparing the null model to models that assume differences in two parameters as in Table 4, the null model is generally more strongly supported by the data than in comparisons of the null model and models that assume differences in only one parameter as in Table 3. In particular, the data provide anecdotal evidence for the null model compared to the model that assumes differences in both the outcome sensitivity and the updating parameter (i.e., model $\mathcal {M}_{\mu _{A}\mu _{a}}$), and moderate evidence for the null model compared to models $\mathcal {M}_{\mu _{A}\mu _{w}}$, $\mathcal {M}_{\mu _{w}\mu _{a}}$, $\mathcal {M}_{\mu _{A}\mu _{c}}$, $\mathcal {M}_{\mu _{A}\mu _{c}}$, and $\mathcal {M}_{\mu _{A}\mu _{w}\mu _{a}}$, respectively. For all of the other model comparisons the Bayes factors are greater than 11, suggesting strong evidence for the null model. Thus, our model selection analyses of the data suggest that it is very unlikely that the intuitive and deliberate groups differ in three or more parameters.

In sum, of all of the models considered, the null model—that is, the model that assumes no differences in the group-level mean parameters of the intuitive and deliberate decision-makers—received most support. In addition, we saw that the evidence for the null model is weakest when the null model is compared to the models that assume differences in the outcome sensitivity and the updating parameter, respectively (i.e., Bayes factors only slightly larger than 1 in favor of the null model), but that the evidence for the null model is strong when it is compared to models that assume that the groups differ on several parameters.

#### **Bayesian latent-mixture modeling**

**Fig. 4:**
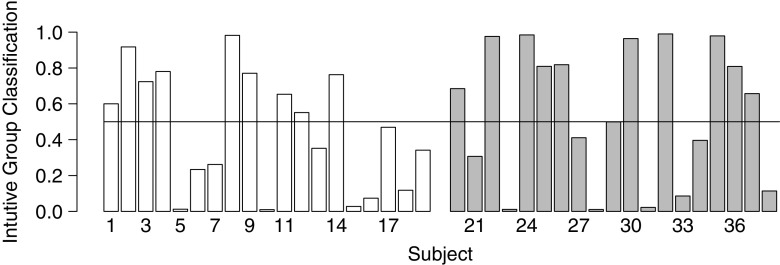
Posterior classification of the individual participants as belonging to the group of intuitive decision-makers based on the latent-mixture analysis. Based to the inventories, participants 1-19 were deliberate decision-makers (i.e., *white bars*), whereas participants 20-38 were intuitive decision-makers (i.e., *grey bars*). The horizontal line represents a posterior classification of .5

Figure 4 shows the posterior means of the *z*_*i*_ variables for each participant. Since these are naturally interpreted as group membership probabilities, a low posterior mean of *z*_*i*_ suggests that the *i* th participant is very likely to belong to the group of deliberate decision-makers, whereas a large value suggests that that participant is very likely to belong to the group of intuitive decision makers. According to the group membership established with the decision-style inventory, participants 1–19 were classified as deliberate decision-makers (i.e., unfilled bars), whereas participants 20–38 were classified as intuitive decision-makers (i.e., grey bars). The horizontal line represents a posterior classification probability of 0.5.

If the self-reported deliberate versus intuitive decision style has a sizeable impact on IGT performance, the latent-mixture model should make inferences consistent with the group membership following from the decision-style inventory. Specifically, for participants 1–19 the posterior mean of the *z*_*i*_ variable should be below the horizontal line, whereas it should be above this line for participants 20–38. However, it is evident in Fig. 4 that the group membership inferred from the latent-mixture modeling analysis does not coincide with the ground truth distinction between intuitive and deliberate decision-makers. Thus, there is strong evidence that the model and data do not distinguish the participants into the groups suggested by the self-report decision-style inventory.

## Discussion

We presented a Bayesian approach for analyzing whether two groups differ in their behavior on the IGT and for using cognitive models to test whether their behavior is driven by different psychological processes. For the latter goal, we used three complementary Bayesian analyses to “triangulate” the research question: hierarchical parameter estimation, Bayes factor model comparison, and latent-mixture modeling (see also Lee et al., 2015).

We illustrated this Bayesian approach with a comparison of the card selection behavior on the IGT of decision-makers who report to prefer an intuitive versus a deliberate decision style. This comparison is interesting because Bechara et al. (1997) proposed that intuitive, affective processes are important for good performance on this task. In addition, although people who report a preference for intuitive versus deliberate decision styles have been found to show differences in several decision tasks, such as valuation of consumer items and monetary lotteries (Schunk & Betsch, 2006; Betsch & Kunz, 2008; see also Phillips et al., 2016), it had yet to be investigated whether such differences generalize to complex decision-making as measured with the IGT.

The application of our Bayesian approach revealed that, at the behavioral level, intuitive and deliberate decision-makers show similar deck preferences on the IGT. All of the three Bayesian modeling analyses suggested that similar cognitive processes drive performance of intuitive and deliberate decision-makers on the IGT. The fact that the three different ways of formalizing the basic research question resulted in consistent findings permits stronger conclusions than could be made based on any one approach alone (Lee et al., 2015).

### Methodological contribution

Even though the Bayes factor is “the standard Bayesian solution to the hypothesis testing and model selection problems” (Lewis and Raftery, 1997; p. 648), to our knowledge this is the first time that Bayes factors have been derived to compare not only the behavioral performance of two groups (i.e., by means of repeated-measures ANOVA), but also to investigate whether two groups differ in PVL-Delta model parameters (i.e., by means of the product space method), and that a latent-mixture extension has been applied to an IGT model. We believe that the use of these methods will advance the study of group differences on the IGT for several reasons. Using our Bayesian suite of analyses we can draw more valid and profound inferences about our research question, many of which are not either possible in the frequentist framework. First, a fundamental difference is that the Bayesian approach allows us to assign probabilities to parameters and hypotheses—a possibility that is in line with researchers’ interests typically *not* concerning the probability of encountering data at least as extreme as those that were observed, given that the null hypothesis is true and the sample was generated according to a specific intended procedure (Lee & Wagenmakers, 2005; Wetzels et al., 2011). Consequently, using the Bayes factor we can infer whether the data are informative enough to draw strong conclusions, and, in the case of informative data, we can infer the probability of the data under the null hypothesis relative to the alternative hypothesis (for more advantages on the Bayesian approach see for example Rouder et al., 2009; Wagenmakers, 2007; Wagenmakers et al., 2008). This is an important advantage of the Bayesian approach, especially given many non-significant results that have been reported in IGT research (see extensive reviews by Sevy et al., 2007; Toplak et al., 2010). From such non-significant results of frequentist tests, one can only conclude that the null hypothesis cannot be rejected. Such a conclusion is clearly less insightful than conclusions allowed for by the Bayes factor.

Second, our suite of methods benefits from the property of the Bayes factor of implementing the tradeoff between a model’s goodness-of-fit and parsimony in a manner that is more comprehensive than that used by the current alternatives. In particular, the Bayes factor coherently and completely discounts model complexity because it considers three dimensions of complexity: (1) the number of free parameters, (2) the functional form of the model, and (3) the extension of the parameter space (Busemeyer et al., in press; Myung & Pitt, 1997), whereas popular alternatives consider only the first dimension (Ahn et al., 2014; Schwarz, 1978; Spiegelhalter et al., 2002).

Third, our suite of methods augments current standard frequentist methods to analyze group differences on the IGT. Our methods rely on more reliable parameter inference (Ahn et al., 2011; Scheibehenne & Pachur, 2015; Shiffrin et al., 2008; Wetzels et al., 2010), they incorporate both commonalities and differences between participants of one group (Navarro et al., 2006; Rouder & Lu, 2005; Rouder et al., 2005; Rouder et al., 2008), and they can be used to quantify evidence for the null hypothesis (for further advantages of the Bayesian approach compared to classical hypothesis testing, see Berger & Delampady, 1987; Edwards et al., 1963; Johnson, 2013; Pocock, 1977; Reboussin *et al*. 2000; Sellke et al., 2001). In addition, the Bayesian approach allows for a straightforward extension of cognitive models to infer group membership—a possibility that we demonstrated with our latent-mixture model. To our knowledge, latent-mixture models to infer group membership from parameters of reinforcement-learning models using a frequentist approach (e.g., using least-squares fitting and maximum likelihood), have not yet been developed. This illustrates that our Bayesian suite of analyses can be used to answer more manifold research questions.

### Self-reported decision styles and decision behavior

Much research has developed and applied reliable self-report instruments for assessing differences between decision-makers in their tendency to rely on the intuitive and the deliberate system (Betsch, 2004; Betsch & Iannello 2010; Pacini & Epstein, 1999). In a recent meta-analysis, that mainly encompassed reasoning and judgment tasks, Phillips et al. (2016) concluded that individual differences in decision style impacts decision-making, but that the particular impact varies considerably across different decision paradigms. In order to investigate to what extent the conclusions of Phillips et al. (2016) also hold for the IGT, we rigorously compared the IGT performance of decision-makers with an intuitive or deliberate decision style. Our results find no evidence that a person’s self-reported preference for an intuitive and a deliberate decision style has a substantial bearing on IGT performance. This result is interesting because the notion of an intuitive decision style is conceptually related to the somatic maker hypothesis, according to which a stronger reliance on an intuitive decision mode results in better IGT performance because of the crucial role of the emotional, intuitive system for learning to make good decisions on the IGT (Damasio, 1994).

There are (at least) two ways to interpret this lack of an association between self-reported preference for a intuitive versus deliberate decision style and IGT performance. First, it is possible that IGT performance does not tap substantially into the affective signals that decision-makers with an intuitive decision style report to pay attention to. This view would provide a challenge to the somatic marker hypothesis, which predicts a strong contribution of affect to IGT performance (for further critical discussion and evidence, see, e.g., Dunn et al., 2006; Tomb et al., 2002). Second, it may be that the dissociation reflects that, similar as for measures of self-reported impulsivity and actual behavior (Janssen et al., 2015), task-specific factors override more general preferences for a particular approach to solve a decision task. That is, the weak association between decision style and IGT performance may be due to the way decision styles are typically assessed. While standard decision-style inventories tap into decision-making in a rather abstract and domain-general fashion, there is indication for considerable domain-specificity of decision style (Pachur & Spaar, 2015). As a consequence, a person’s domain-general decision style might only weakly predict her decision style in a financial risk-taking task such as the IGT. In general, this interpretation is consistent with the view that people can flexibly adapt their decision-making processes to characteristics of the task (e.g., Gigerenzer et al., 2011; Payne et al., 1993).

Why did Phillips et al. (2016) find evidence for an association between decision style and decision-making performance, whereas we failed to find such an association in the context of the IGT? Phillips et al. (2016) obtained the strongest benefit of a deliberate decision style in the context of inductive reasoning tasks, where often one particular suggestive response has to be overridden; the strongest benefit of an intuitive decision style was obtained for tasks involving the generation of alternatives or ideas. The IGT, by contrast, involves a careful deliberation and learning of the options’ payoff from experience (cf. Schonberg et al., 2011), and all the options are explicitly given in the task. Potentially, the complex and engaging nature of the IGT, tapping into multiple psychological processes (such as motivation, memory, and response consistency) might thus override the influence of a person’s decision style.

If decision style is not associated with performance on the IGT, what other factors might account for individual variability commonly observed in this task? One possibility is that more task-specific capacities such as working memory, intelligence, and inhibition play a crucial role. On the other hand, although some studies have indeed found IGT performance to be linked to variables such as working memory, inhibition, intelligence, and personality (e.g., Crone et al., 2003; Demaree et al., 2010; Franken & Muris, 2005; Suhr & Tsanadis, 2007), such links seem to emerge inconsistently and are, overall, rather weak (e.g., Dunn et al., 2006; Toplak et al., 2010).

### Conclusions

We proposed a set of Bayesian analyses for comparing IGT performance between two groups. The application of these techniques to compare decision-makers with a deliberate or an intuitive decision style showed not only that both groups of decision-makers perform similarly on the IGT, but also that their performance is driven by similar cognitive processes. Our refined analysis approach could easily be adapted to other decision-making tasks and cognitive models of behavior on those tasks. All of the relevant code is available online, and all of the required programs are free to download. Due to the advantages of Bayesian analyses, we encourage using our proposed methods to investigate group differences in IGT data or in similar decision-making tasks.

## Appendix A: Experiment

### Material

#### **Iowa Gambling Task**

The IGT was administered as a computerized task, based on the original version of Bechara et al. (1994).^9^ On the computer screen, four decks of cards were presented, labeled “A”, “B”, “C”, and “D”. Participants were initially given a (hypothetical) loan of +2000 Swiss Francs (CHF). They were instructed to consecutively choose among the decks in such a way that they maximize their long-term net outcome (cf. Bechara et al., 1994; Bechara et al., 1997). At each trial, a deck could be selected by clicking on it. Each choice resulted in a draw of a card from the chosen deck, and feedback on the gains as well as the losses (if any) associated with the card, and the running tally. The trials were self-paced and the task stopped after 100 trials.

#### **Measurement of decision style**

To measure individual participants’ decision style, we used an inventory complied by Betsch and Iannello (in preparation), whose subscales are taken from five different questionnaires: the Rational-Experiential Inventory (REI; Pacini and Epstein, 1999), the Preference for Intuition and Deliberate Scale (PID; Betsch, 2004), the General Decision Making Style (GDMS; Scott & Bruce, 1995) questionnaire, the Cognitive Style Indicator (CoSI; Cools & van den Broeck, 2007), and the Perceived Modes of Processing Inventory (PMPI; Burns & D’Zurilla, 1999). All of these questionnaires measure a person’s tendency to rely on an intuitive and a deliberate decision mode on two separate bipolar subscales. For instance, participants are presented with statements such as “My feelings play an important role in my decisions.” (intuition subscale of the PID), or “Before making decisions, I first think them through.” (deliberation subscale of the PID). At each item, participants are asked to indicate the extent to which the statement represents their opinion (on a scale from 1 = very much disagree to 7 = very much agree). The original versions of the REI, PID and the GDMS contain items that include the term “intuition”. Betsch & Iannello (2010) argued that this might activate different concepts across people. These items were therefore excluded from Betsch and Iannello’s (in preparation) compiled inventory. Altogether, the questionnaire consisted of 70 items from 12 subscales (Table 5). As described in more detail below, we distinguished intuitive and deliberate decision-makers based on their total scores on these subscales.

**Table 5:** Seventy questionnaire items with their respective subscale from Betsch and Iannello’s (in preparation) compiled inventory

Item	Subscale
I quickly do the right thing when coping because I’ve often faced almost the same thing before.	PMPI 6
I make definite engagements, and I follow up meticulously.	CoSi-p 6
I prefer clear structures to do my job.	CoSi-p 4
With most decisions it makes sense to completely rely on your feelings.	PID-i 2
I don’t like to have to do a lot of thinking.	REI-re 3
Developing a clear plan is very important to me.	CoSi-p 1
I generally make snap decisions.	GDMS-s 1
I often make impulsive decisions.	GDMS-s 4
I like detailed action plans.	CoSi-p 3
Using logic usually works well for me in figuring out problems in my life.	REI-ra 9
Using my gut feelings usually works well for me in figuring out problems in my life.	REI-ea 1
I prefer making detailed plans rather than leaving things to chance.	PID-d 3
I hardly ever go wrong when I listen to my deepest gut feelings to find an answer.	REI-ea 6
I’ve had enough experience to just know what I need to do to cope most of the time without trying to figure it out every time.	PMPI 3
I prefer well-prepared meetings with a clear agenda and strict time management.	CoSi-p 5
I can usually feel when a person is right or wrong, even if I can’t explain how I know.	REI-ea 8
I like to analyze problems.	CoSi-k 2
Knowing the answer without having to understand the reasoning behind it is good enough for me.	REI-re 9
I enjoy intellectual challenges.	REI-re 2
I think about a decision particularly carefully if I have to justify it.	PID-d 5
I always want to know what should be done when.	CoSi-p 2
I tend to use my heart as a guide for my actions.	REI-ee 4
I double-check my information sources to be sure I have the right facts before making decisions.	GDMS-d 2
I study every problem until I understand the underlying logic.	CoSi-k 4
My decision-making requires careful thought.	GDMS-d 4
When I have a problem I first analyze the facts and details before I decide.	PID-d 6
Thinking is not my idea of an enjoyable activity.	REI-re 5
I rely mostly on my past experience to find a way to cope.	PMPI 8
Before making decisions I usually think about the goals I want to achieve.	PID-d2
I prefer complex to simple problems.	REI-re 6
I generally don’t depend on my feelings to help me make decisions.	REI-ee 3
I usually have clear, explainable reasons for my decisions.	REI-ra 10
Most of the time, I use the same method to cope.	PMPI 7
If an approach works I use it again and again so I don’t have to come up with a new one for each stressful situation I face.	PMPI 2
I want to have a full understanding of all problems.	CoSi-k 1
I am not a very analytical thinker.	REI-ra 3
I make detailed analysis.	CoSi-k 3
I have a logical mind.	REI-ra 7
Reasoning things out carefully is not one of my strong points.	REI-ra 4
I am often aware of how to cope with a stressful situation even before I review all its aspects.	PMPI 1
I think before I act.	PID-d 7
I prefer emotional people.	PID-i 5
Thinking hard and for a long time about something gives me little satisfaction.	REI-re 7
The right way to cope usually comes to mind almost immediately.	PMPI 4
I have no problem thinking things through carefully.	REI-ra 8
I trust my initial feelings about people.	REI-ea 3
When it comes to trusting people, I can usually rely on my gut feelings.	REI-ea 4
I enjoy solving problems that require hard thinking.	REI-re 4
When I make a decision, it is more important for me to feel the decision is right than to have a rational reason for it.	GDMS-i 3
When I make a decision, I trust my innermost feelings and reactions.	GDMS-i 4
I typically figure out the way to cope swiftly.	PMPI 5
Learning new ways to think would be very appealing to me.	REI-re 10
A good task is a well-prepared task.	CoSi-p 7
Before making decisions I first think them through.	PID-d 1
I rarely need to mull things over; how to cope usually becomes quickly apparent.	PMPI 9
I enjoy thinking in abstract terms.	REI-re 8
If I were to rely on my gut feelings, I would often make mistakes.	REI-ea 5
I plan my important decisions carefully.	GDMS-d 1
I prefer drawing conclusions based on my feelings, my knowledge of human nature, and my experience of life.	PID-i 3
I make decisions in a logical and systematic way.	GDMS-d 3
When making a decision, I consider various options in terms of a specific goal.	GDMS-d 5
I don’t think it is a good idea to rely on one’s intuition for important decisions.	REI-ee 2
I believe in trusting my hunches.	REI-ea 2
My feelings play an important role in my decisions.	PID-i 4
I try to avoid situations that require thinking in depth about something.	REI-re 1
When making decisions, I do what seems natural at the moment.	GDMS-s 5
I often make decisions on the spur of the moment.	GDMS-s 2
I like emotional situations, discussions and movies.	PID-i 6
I make quick decisions.	GDMS-s 3
I generally make decisions that feel right to me.	GDMS-i 2

Note. The second column indicates the instrument from which the corresponding item was taken. GDMS = General Decision Making Style inventory (Scott & Bruce, 1995); PID = Preference for Intuition and Deliberation scale (Betsch, 2004); REI = Rational- Experiential Inventory (Pacini & Epstein, 1999); PMPI = Perceived Modes of Processing Inventory (Burns & D’Zurilla, 1999); CoSI = Cognitive Style Indicator (Cools & van den Broeck, 2007). p = planning, i = intuition, re = rational engagement, ra = rational ability, ee = experiential engagement, ea = experiential ability, k = knowledge, d = deliberation.

### Procedure

Participants completed the experiment individually. They signed an informed consent form and started the experiment with the IGT, followed by demographic questions and a computerized version of the decision-style inventory. Then they were thanked, debriefed, and received course credits or a flat fee of 7.50 CHF—a decision that had to be made before the experiment—as well as a performance-contingent bonus from their IGT performance (specifically, final IGT score/1000 * 1.5 CHF).

### Decision style

We first determined for each participant the mean score on each of the 12 subscales of the decision-style inventory compiled by Betsch and Iannello (in preparation). Table 6 shows that all subscales had acceptable levels of internal reliability, except for the experiential engagement subscale of the REI. However, we decided to keep that subscale in our analyses because excluding it did not lead to different conclusions in the subsequent analyses. Based on each participant’s mean score on each of the 12 subscales, we then conducted a principal component analysis with rotation based on the varimax method using the principal() function of the R package psych. The Kaiser criterion suggested a three-factor solution, which accounted for 70% of the total variance. Table 6 reports the factor loadings of the 12 subscales on these three factors. On the first factor the subscales capturing a deliberate, rational, and planned decision style showed consistently high loadings (deliberation factor). The second factor had consistently high loadings for the subscales capturing an intuitive and experiential decision style (intuition factor). The third factor had high loadings for the subscale capturing spontaneous decision-making (spontaneity factor). Individually, the three factors accounted for 33.9%, 22.4%, and 13.4% of the variance, respectively.

**Table 6:** Three-factor solution of the principal component analysis. Also reported are Cronbach’s *α* as a measure of the reliability of each subscale

Subscale	Factor		
	Deliberation	Intuition	Spontaneity
Deliberation (GDMS) (*α* = .80)	**.890**	.008	–.184
Deliberation (PID) (*α* = .74)	**.875**	–.171	–.094
Knowing (CoSI) (*α* = .79)	**.873**	–.068	–.126
Rational ability (REI) (*α* = .77)	**.820**	–.148	.176
Rational engagement (REI) (*α* = .81)	**.659**	–.106	–.145
Planning (CoSI) (*α* = .70)	**.601**	–.326	.130
Experiental ability (REI) (*α* = .84)	.059	**.870**	.165
Intuition (GDMS) (*α* = .69)	–.121	**.850**	.056
Intuition (PID) (*α* = .79)	–.150	**.809**	.018
Experiential engagement (REI) (*α* = .41)	–.311	**.604**	.033
Automatic (PMPI) (*α* = .77)	.115	.126	**.897**
Spontaneous (GDMS) (*α* = .73)	–.354	.076	**.805**

Following previous research (Betsch & Kunz, 2008), we classified participants as intuitive if they had a factor score above the median of the intuition factor and, at the same time, a factor score below the median of the deliberation factor; participants with the opposite pattern were classified as deliberate.^10^ This classification scheme yielded 19 participants in the intuitive group and 19 participants in the deliberate group. Thirty-two participants thus remained unclassified and were excluded from the analyses presented in the main article.

## Appendix B: Obtaining Bayes Factors with the Product Space Method

In this section we describe how we obtained the Bayes factor with the product space method (Carlin & Chib, 1995; Lodewyckx et al., 2011). The Bayes factor BF_12_ is defined as the change from prior model odds ${p(\mathcal {M}_{1})}/{p(\mathcal {M}_{2})}$ of two models, $\mathcal {M}_{1}$ and $\mathcal {M}_{2}$, to posterior model odds ${p(\mathcal {M}_{1}\mid D)}/{p(\mathcal {M}_{2} \mid D)}$ brought about by the data *D*:

5 $$ \underbrace{\frac{p(\mathcal{M}_{1}\mid D)}{p(\mathcal{M}_{2} \mid D)}}_{\text{Posterior model odds}} = \underbrace{\frac{p(\mathcal{M}_{1})}{p(\mathcal{M}_{2})}}_{\text{Prior model odds}} \times \; \; \; \; \underbrace{\frac{m(D \mid \mathcal{M}_{1})}{m(D \mid \mathcal{M}_{2})}}_{\text{Bayes factor}} $$

For all but the simplest models the Bayes factor cannot be derived analytically. We therefore need a method to approximate the Bayes factor. One such method is the product space method (for alternative methods such as reversible jump, see Green, 2003; Sisson, 2005, and for importance sampling, see Hammersley & Handscomb, 1964; Steingroever et al., 2016; Vandekerckhove et al., 2015). The product space method is a transdimensional Markov chain Monte Carlo (MCMC) method, a method that aims to estimate the posterior model odds for chosen prior model odds (see Eq. 5). This method requires the construction of a “supermodel” encompassing the models to be compared. This “supermodel” is a hierarchical combination of the models to be compared. The hierarchical combination is achieved by a model index that measures the proportion of times that either model is visited to account for the observed data. The prior of the model index corresponds to the prior model odds (i.e., specified before the analysis), and the posterior of the model index corresponds to the posterior model odds. The posterior model index can be estimated by MCMC posterior sampling methods. We can therefore estimate the posterior probability of model $\mathcal {M}_ k$ using:

6 $$ \hat{p}(\mathcal{M}_ k \mid D) = \frac{\text{Number of occurrences of }\mathcal{M}_ k}{\text{Total number of iterations}}. $$

The posterior model probability quantifies the relative plausibility for model $\mathcal {M}_ k$ given the prior model probability and the evidence from the data (Berger & Molina 2005). Given the estimated posterior model probabilities of two different models, we can estimate the Bayes factor using Eq. 5 because the prior model odds are known (i.e., specified before the analysis).

## Footnotes

1 For philosophical and fundamental differences between the Bayesian and frequentist approach see Lee and Wagenmakers (2005) and Wetzels et al. (2011). Most crucially, these articles contrast the Bayesian approach with the frequentist approach by arguing that the two allow for completely different kinds of inferences. The frequentist approach, on the one hand, provides the probability of encountering data at least as extreme as those that were observed, given that the null hypothesis is true and the sample was generated according to a specific intended procedure. The Bayesian approach, on the other hand, provides the relative plausibility of the hypotheses under consideration after having observed the data. However, only the latter kind of inference is useful for the research question we investigate in this article, but also for research questions in psychology more generally. This underscores the superiority of the Bayesian approach. Additional discussions can be found in Andrews and Baguley (2013), Bayarri et al. (2016), Rouder et al. (2009), Sellke et al. (2001), and Wagenmakers (2007).

2 We initialized the expectancies of each deck *k* to zero, so that *Ev*_*k*_(0) = 0.

3 In total, there are 2^4^ = 16 possible models, one of which is the null model. Therefore, we obtained 15 model comparisons involving a model that assumes differences in at least one group-level mean parameter across the two groups compared to the null model.

4 The discussion can be found here https://groups.google.com/forum/?hl=cs#!searchin/stan-users/reinforcemen/stan-users/TjY3wQqUS2g/cff2lWoRUr0J.

5 Note that our two-group assumption has a clear intuitive-deliberate basis in theory; however, in general, it is also possible to assume more than two groups (see Bartlema et al., 2014).

6 We repeated the analyses presented in this article using an alternative classification which included all participants and simply distinguished between intuitive and deliberate decision-makers based on whether or not their score on the intuition factor was higher than the median. These analyses resulted in qualitatively identical conclusions as the ones presented in this article.

7 The frequentist repeated-measures ANOVA revealed that neither the main effect of decision style (*F*(1, 36) = .404, *p* = .529) nor the interaction between block and decision style (*F*(9, 324) = 1.466, *p* = .159) was significant.

8 The Bayes factors discussed in this article are based on unrounded posterior model probabilities and may therefore slightly differ from Bayes factors calculated using the posterior model probabilities presented in Table 3.

## References

[CR1] Ahn W-Y, Busemeyer JR, Wagenmakers E-J, Stout JC (2008). Comparison of decision learning models using the generalization criterion method. Cognitive Science.

[CR2] Ahn, W.-Y., Haines, N., & Zhang, L (2016). Revealing neuro-computational mechanisms of reinforcement learning and decision-making with the hBayesDM package. bioRxiv. 10.1162/CPSY_a_00002 PMC5869013 29601060

[CR3] Ahn W-Y, Krawitz A, Kim W, Busemeyer JR, Brown JW (2011). A model-based fMRI analysis with hierarchical Bayesian parameter estimation. Journal of Neuroscience Psychology and Economics.

[CR4] Ahn W-Y, Vasilev G, Lee SH, Busemeyer JR, Kruschke JK, Bechara A (2014). Decision-making in stimulant and opiate addicts in protracted abstinence: Evidence from computational modeling with pure users. Frontiers in Psychology.

[CR5] Andrews M, Baguley T (2013). Prior approval: The growth of Bayesian methods in psychology. British Journal of Mathematical and Statistical Psychology.

[CR6] Bark R, Dieckmann S, Bogerts B, Northoff G (2005). Deficit in decision-making in catatonic schizophrenia: An exploratory study. Psychiatry Research.

[CR7] Bartlema A, Lee M, Wetzels R, Vanpaemel W (2014). A Bayesian hierarchical mixture approach to individual differences: Case studies in selective attention and representation in category learning. Journal of Mathematical Psychology.

[CR8] Bayarri MJ, Benjamin DJ, Berger JO, Sellke TM (2016). Rejection odds and rejection ratios: A proposal for statistical practice in testing hypotheses. Journal of Mathematical Psychology.

[CR9] Bechara A, Damasio AR, Damasio H, Anderson SW (1994). Insensitivity to future consequences following damage to human prefrontal cortex. Cognition.

[CR10] Bechara A, Damasio H, Damasio AR, Lee GP (1999). Different contributions of the human amygdala and ventromedial prefrontal cortex to decision-making. Journal of Neuroscience.

[CR11] Bechara A, Damasio H, Tranel D, Anderson SW (1998). Dissociation of working memory from decision-making within the human prefrontal cortex. Journal of Neuroscience.

[CR12] Bechara A, Damasio H, Tranel D, Damasio AR (1997). Deciding advantageously before knowing the advantageous strategy. Science.

[CR13] Bechara A, Tranel D, Damasio H (2000). Characterization of the decision-making deficit of patients with ventromedial prefrontal cortex lesions. Brain.

[CR14] Beitz KM, Salthouse TA, Hasker DP (2014). Performance on the Iowa Gambling Task: From 5 to 89 years of age. Journal of Experimental Psychology: General.

[CR15] Berger, J. O., & Delampady, M. (1987). Testing precise hypotheses. *Statistical Science, 2,* 317–335.

[CR16] Berger JO, Molina G (2005). Posterior model probabilities via path-based pairwise priors. Statistica Neerlandica.

[CR17] Berger JO, Mortera J (1999). Default Bayes factors for nonnested hypothesis testing. Journal of the American Statistical Association.

[CR18] Betsch C (2004). Präferenz für Intuition und Deliberation. Zeitschrift für Differentielle und Diagnostische Psychologie.

[CR19] Betsch C, Iannello P, Glöckner A, Witteman C (2010). Measuring individual differences in intuitive and deliberate decision making styles – A comparison of different measures. Tracing intuition: Recent methods in measuring intuitive and deliberate processes in decision making.

[CR20] Betsch, C., & Iannello, P. (in preparation). A unified scale to assess individual differences in intuition and deliberation (USID).

[CR21] Betsch C, Kunz JJ (2008). Individual strategy preferences and decisional fit. Journal of Behavioral Decision Making.

[CR22] Blair RJR, Colledge E, Mitchell DGV (2001). Somatic markers and response reversal: Is there orbitofrontal cortex dysfunction in boys with psychopathic tendencies?. Journal of Abnormal Child Psychology.

[CR23] Buelow MT, Suhr JA (2009). Construct validity of the Iowa Gambling Task. Neuropsychology Review.

[CR24] Burns LR, D’Zurilla TJ (1999). Individual differences in perceived information processing styles in stress and coping situations: Development and validation of the perceived modes of processing inventory. Cognitive Therapy and Research.

[CR25] Busemeyer JR, Stout J, Finn P, Barch D (2003). Using computational models to help explain decision-making processes of substance abusers. Cognitive and affective neuroscience of psychopathology.

[CR26] Busemeyer JR, Stout JC (2002). A contribution of cognitive decision models to clinical assessment: Decomposing performance on the Bechara gambling task. Psychological Assessment.

[CR27] Busemeyer, J. R., Wang, Z., & Shiffrin, R. M. (in press). Bayesian model comparison favors quantum over standard decision theory account of dynamic inconsistency. *Decision*.

[CR28] Carlin, B. P., & Chib, S. (1995). Bayesian model choice via Markov chain Monte Carlo methods. *Journal of the Royal Statistical Society. Series B (Methodological), 3,* 473–484.

[CR29] Cavedini P, Riboldi G, D’Annucci A, Belotti P, Cisima M, Bellodi L (2002). Decision-making heterogeneity in obsessive–compulsive disorder: Ventromedial prefrontal cortex function predicts different treatment outcomes. Neuropsychologia.

[CR30] Cavedini P, Riboldi G, Keller R, D’annucci A, Bellodi L (2002). Frontal lobe dysfunction in pathological gambling patients. Biological Psychiatry.

[CR31] Cella M, Dymond S, Cooper A, Turnbull OH (2012). Cognitive decision modelling of emotion-based learning impairment in schizophrenia: The role of awareness. Psychiatry Research.

[CR32] Cools, E., & van den Broeck, H. (2007). Development and validation of the cognitive style indicator. *The Journal of Psychology*, *141*, 359–387. 10.3200/jrlp.141.4.359-388 17725071

[CR33] Crone, E. A., Vendel, I., & van der Molen, M. W. (2003). Decision-making in disinhibited adolescents and adults: Insensitivity to future consequences or driven by immediate reward? *Personality and Individual Differences*, *35*, 1625–1641.

[CR34] Dai J, Kerestes R, Upton DJ, Busemeyer JR, Stout JC (2015). An improved cognitive model of the Iowa and Soochow gambling tasks with regard to model fitting performance and tests of parameter consistency. Frontiers in Psychology.

[CR35] Damasio, A. R. (1994). *Descartes’ error: Emotion reason and the human brain*. New York: Avon.

[CR36] Damasio, A. R., Tranel, D., & Damasio, H (1991). Somatic markers and the guidance of behavior: Theory and preliminary testing. In Levin, H, Eisenberg, H., & Benton, A. E. (Eds.), *Frontal lobe function and dysfunction* (pp. 217–229). New York: Oxford University Press.

[CR37] Davis, C., Fox, J., Patte, K., Curtis, C., Strimas, R., Reid, C., et al. (2008). Education level moderates learning on two versions of the Iowa Gambling Task. *Journal of the International Neuropsychological Society*, *14*, 1063–1068. 10.1017/S1355617708081204 18954486

[CR38] Demaree HA, Burns KJ, DeDonno MA (2010). Intelligence, but not emotional intelligence, predicts Iowa Gambling Task performance. Intelligence.

[CR39] Dunn BD, Dalgleish T, Lawrence AD (2006). The somatic marker hypothesis: A critical evaluation. Neuroscience & Biobehavioral Reviews.

[CR40] Edwards W, Lindman H, Savage LJ (1963). Bayesian statistical inference for psychological research. Psychological Review.

[CR41] Epstein S, Pacini R, Denes-Raj V, Heier H (1996). Individual differences in intuitive–experiential and analytical–rational thinking styles. Journal of Personality and Social Psychology.

[CR42] Escartin, G., Junqué, C., Juncadella, M., Gabarrós, A., de Miquel, M. A., & Rubio, F. (2012). Decision-making impairment on the Iowa Gambling Task after endovascular coiling or neurosurgical clipping for ruptured anterior communicating artery aneurysm. *Neuropsychology*, *26*, 172–180. 10.1037/a0024336 22251310

[CR43] Franken IH, Muris P (2005). Individual differences in decision-making. Personality and Individual Differences.

[CR44] Fridberg DJ, Queller S, Ahn W-Y, Kim W, Bishara AJ, Busemeyer JR (2010). Cognitive mechanisms underlying risky decision-making in chronic cannabis users. Journal of Mathematical Psychology.

[CR45] Gelman A, Rubin D (1992). Inference from iterative simulation using multiple sequences. Statistical Science.

[CR46] Gigerenzer G, Hertwig R, Pachur T (2011). Heuristics: The foundations of adaptive behavior.

[CR47] Green, P. J. (2003). Trans-dimensional Markov chain Monte Carlo. In Green, P. J., Hjort, N. L., & Richardson, S. (Eds.), *Highly structured stochastic systems.* Oxford University Press.

[CR48] Hammersley JM, Handscomb DC (1964). Monte Carlo methods.

[CR49] Harman JL (2011). Individual differences in need for cognition and decision-making in the Iowa Gambling Task. Personality and Individual Differences.

[CR50] Hoffman MD, Gelman A (2014). The no-U-turn sampler: Adaptively setting path lengths in Hamiltonian Monte Carlo. Journal of Machine Learning Research.

[CR51] Horn SS, Pachur T, Mata R (2015). How does aging affect recognition-based inference? A hierarchical Bayesian modeling approach. Acta Psychologica.

[CR52] Janssen T, Larsen H, Peeters M, Boendermaker WJ, Vollebergh WA, Wiers RW (2015). Do online assessed self-report and behavioral measures of impulsivity-related constructs predict onset of substance use in adolescents?. Addictive Behaviors Reports.

[CR53] JASP Team (2015). JASP (Version 0.7) [Computer software].

[CR54] Jeffreys H (1961). Theory of probability.

[CR55] Johnson VE (2013). Revised standards for statistical evidence. Proceedings of the National Academy of Sciences.

[CR56] Kass RE, Raftery AE (1995). Bayes factors. Journal of the American Statistical Association.

[CR57] Lee MD, Lodewyckx T, Wagenmakers E-J, Raaijmakers J R, Criss A, Goldstone R, Nosofsky R, Steyvers M (2015). Three Bayesian analyses of memory deficits in patients with dissociative identity disorder. Cognitive modeling in perception and memory: A Festschrift for Richard M. Shiffrin.

[CR58] Lee MD, Wagenmakers E-J (2005). Bayesian statistical inference in psychology: Comment on Trafimow (2003). Psychological Review.

[CR59] Lee MD, Wagenmakers E-J (2013). Bayesian modeling for cognitive science: A practical course.

[CR60] Lejarraga T, Pachur T, Frey R, Hertwig R (2016). Decisions from experience: From monetary to medical gambles. Journal of Behavioral Decision Making.

[CR61] Lewis SM, Raftery AE (1997). Estimating Bayes factors via posterior simulation with the Laplace-Metropolis estimator. Journal of the American Statistical Association.

[CR62] Lodewyckx T, Kim W, Lee MD, Tuerlinckx F, Kuppens P, Wagenmakers E-J (2011). A tutorial on Bayes factor estimation with the product space method. Journal of Mathematical Psychology.

[CR63] Luce RD (1959). Individual choice behavior: A theoretical analysis.

[CR64] Maia TV, McClelland JL (2004). A reexamination of the evidence for the somatic marker hypothesis: What participants really know in the Iowa Gambling Task. Proceedings of the National Academy of Sciences of the United States of America.

[CR65] Martino DJ, Bucay D, Butman JT, Allegri RF (2007). Neuropsychological frontal impairments and negative symptoms in schizophrenia. Psychiatry Research.

[CR66] Myung IJ, Pitt MA (1997). Applying Occam’s razor in modeling cognition: A Bayesian approach. Psychonomic Bulletin & Review.

[CR67] Navarro DJ, Griffiths TL, Steyvers M, Lee MD (2006). Modeling individual differences using Dirichlet processes. Journal of Mathematical Psychology.

[CR68] Newell BR, Shanks DR (2014). Unconscious influences on decision-making: A critical review. Behavioral and Brain Sciences.

[CR69] Pachur T, Olsson H (2012). Type of learning task impacts performance and strategy selection in decision-making. Cognitive Psychology.

[CR70] Pachur T, Spaar M (2015). Domain-specific preferences for intuition and deliberation in decision-making. Journal of Applied Research in Memory and Cognition.

[CR71] Pacini R, Epstein S (1999). The relation of rational and experiential information processing styles to personality, basic beliefs, and the ratio-bias phenomenon. Journal of Personality and Social Psychology.

[CR72] Payne JW, Bettman JR, Johnson EJ (1993). The adaptive decision maker.

[CR73] Phillips WJ, Fletcher JM, Marks AD, Hine DW (2016). Thinking styles and decision-making: A meta-analysis. Psychological Bulletin.

[CR74] Pocock SJ (1977). Group sequential methods in the design and analysis of clinical trials. Biometrika.

[CR75] R Core Team (2015). R: A language and environment for statistical computing. Vienna, Austria.

[CR76] Reboussin DM, DeMets DL, Kim K, Lan KG (2000). Computations for group sequential boundaries using the Lan-Demets spending function method. Controlled Clinical Trials.

[CR77] Rouder JN (2014). Optional stopping: No problem for Bayesians. Psychonomic Bulletin & Review.

[CR78] Rouder JN, Lu J (2005). An introduction to Bayesian hierarchical models with an application in the theory of signal detection. Psychonomic Bulletin & Review.

[CR79] Rouder JN, Lu J, Morey RD, Sun D, Speckman PL (2008). A hierarchical process-dissociation model. Journal of Experimental Psychology: General.

[CR80] Rouder JN, Lu J, Speckman P, Sun D, Jiang Y (2005). A hierarchical model for estimating response time distributions. Psychonomic Bulletin & Review.

[CR81] Rouder JN, Morey RD, Speckman PL, Province JM (2012). Default Bayes factors for ANOVA designs. Journal of Mathematical Psychology.

[CR82] Rouder JN, Speckman PL, Sun D, Morey RD, Iverson G (2009). Bayesian t tests for accepting and rejecting the null hypothesis. Psychonomic Bulletin & Review.

[CR83] Scheibehenne B, Pachur T (2015). Using Bayesian hierarchical parameter estimation to assess the generalizability of cognitive models of choice. Psychonomic Bulletin & Review.

[CR84] Schonberg T, Fox CR, Poldrack RA (2011). Mind the gap: Bridging economic and naturalistic risk-taking with cognitive neuroscience. Trends in Cognitive Sciences.

[CR85] Schunk D, Betsch C (2006). Explaining heterogeneity in utility functions by individual differences in decision modes. Journal of Economic Psychology.

[CR86] Schwarz G (1978). Estimating the dimension of a model. The Annals of Statistics.

[CR87] Scott SG, Bruce RA (1995). Decision-making style: The development and assessment of a new measure. Educational and Psychological Measurement.

[CR88] Sellke T, Bayarri M, Berger JO (2001). Calibration of p values for testing precise null hypotheses. The American Statistician.

[CR89] Sevy S, Burdick KE, Visweswaraiah H, Abdelmessih S, Lukin M, Yechiam E (2007). Iowa Gambling Task in schizophrenia: A review and new data in patients with schizophrenia and co-occurring cannabis use disorders. Schizophrenia Research.

[CR90] Shiffrin RM, Lee MD, Kim W, Wagenmakers E-J (2008). A survey of model evaluation approaches with a tutorial on hierarchical Bayesian methods. Cognitive Science.

[CR91] Sisson SA (2005). Transdimensional Markov chains: A decade of progress and future perspectives. Journal of the American Statistical Association.

[CR92] Spiegelhalter, D. J., Best, N. G., Carlin, B. P., & van der Linde, A (2002). Bayesian measures of model complexity and fit. *Journal of the Royal Statistical Society: Series B (Statistical Methodology)*, *64*, 583–639.

[CR93] Stan Development Team (2014a). RStan: The R interface to Stan, version 2.5.0. Retrieved from http://mc-stan.org/rstan.html

[CR94] Stan Development Team (2014b). Stan: A C++ library for probability and sampling, version 2.5.0.

[CR95] Stan Development Team (2014c). Stan modeling language users guide and reference manual, version 2.5.0.

[CR96] Steingroever H, Davis H, Fridberg DJ, Horstmann A, Kjome KL, Kumari V (2015). Data from 617 healthy participants performing the Iowa Gambling Task: A “many labs” collaboration. Journal of Open Psychology Data.

[CR97] Steingroever H, Wetzels R, Horstmann A, Neumann J, Wagenmakers E-J (2013). Performance of healthy participants on the Iowa Gambling Task. Psychological Assessment.

[CR98] Steingroever, H., Wetzels, R., & Wagenmakers, E.-J. (2013a). A comparison of reinforcement-learning models for the Iowa Gambling Task using parameter space partitioning. *The Journal of Problem Solving*, 5, Article 2.

[CR99] Steingroever H, Wetzels R, Wagenmakers E-J (2013). Validating the PV,L-Delta model for the Iowa Gambling Task. Frontiers in Psychology.

[CR100] Steingroever H, Wetzels R, Wagenmakers E-J (2014). Absolute performance of reinforcement-learning models for the Iowa Gambling Task. Decision.

[CR101] Steingroever H, Wetzels R, Wagenmakers E-J (2016). Bayes factors for reinforcement-learning models of the Iowa Gambling Task. Decision.

[CR102] Suhr JA, Tsanadis J (2007). Affect and personality correlates of the Iowa Gambling Task. Personality and Individual Differences.

[CR103] Tomb I, Hauser M, Deldin P, Caramazza A (2002). Do somatic markers mediate decisions on the gambling task?. Nature Neuroscience.

[CR104] Toplak M, Sorge G, Benoit A, West R, Stanovich K (2010). Decision-making and cognitive abilities: A review of associations between Iowa Gambling Task performance, executive functions, and intelligence. Clinical Psychology Review.

[CR105] Turnbull OH, Evans CE, Bunce A, Carzolio B, O’Connor J (2005). Emotion-based learning and central executive resources: An investigation of intuition and the Iowa Gambling Task. Brain and Cognition.

[CR106] Tversky A, Kahneman D (1992). Advances in prospect theory: Cumulative representation of uncertainty. Journal of Risk and Uncertainty.

[CR107] Vandekerckhove J, Matzke D, Wagenmakers E-J, Busemeyer J, Townsend J, Wang Z J, Eidels A (2015). Model comparison and the principle of parsimony. Oxford handbook of computational and mathematical psychology.

[CR108] Wagenmakers E-J (2007). A practical solution to the pervasive problems of p values. Psychonomic Bulletin & Review.

[CR109] Wagenmakers E-J, Lee M, Lodewyckx T, Iverson GJ, Hoijtink H, Klugkist I, Boelen P A (2008). Bayesian versus frequentist inference. Bayesian evaluation of informative hypotheses.

[CR110] Wagenmakers E-J, Lodewyckx T, Kuriyal H, Grasman R (2010). Bayesian hypothesis testing for psychologists: A tutorial on the Savage–Dickey method. Cognitive Psychology.

[CR111] Wetzels R, Matzke D, Lee MD, Rouder JN, Iverson GJ, Wagenmakers E-J (2011). Statistical evidence in experimental psychology an empirical comparison using 855 t tests. Perspectives on Psychological Science.

[CR112] Wetzels R, Raaijmakers JG, Jakab E, Wagenmakers E-J (2009). How to quantify support for and against the null hypothesis: A flexible WinB,UGS implementation of a default Bayesian t test. Psychonomic Bulletin & Review.

[CR113] Wetzels R, Vandekerckhove J, Tuerlinckx F, Wagenmakers E-J (2010). Bayesian parameter estimation in the Expectancy Valence model of the Iowa Gambling Task. Journal of Mathematical Psychology.

[CR114] Wood S, Busemeyer J, Koling A, Cox CR, Davis H (2005). Older adults as adaptive decision-makers: Evidence from the Iowa Gambling Task. Psychology and Aging.

[CR115] Worthy, D. A., Pang, B., & Byrne, K. A. (2013). Decomposing the roles of perseveration and expected value representation in models of the Iowa Gambling Task. *Frontiers in Psychology*, 4, 640. 10.3389/fpsyg.2013.00640 PMC3786232 24137137

[CR116] Yechiam E, Hayden EP, Bodkins M, O’Donnell BF, Hetrick WP (2008). Decision making in bipolar disorder: A cognitive modeling approach. Psychiatry Research.

[CR117] Yechiam E, Kanz JE, Bechara A, Stout JC, Busemeyer JR, Altmaier EM (2008). Neurocognitive deficits related to poor decision-making in people behind bars. Psychonomic Bulletin & Review.

